# Hypertonic dextrose injections (prolotherapy) in the treatment of symptomatic knee osteoarthritis: A systematic review and meta-analysis

**DOI:** 10.1038/srep25247

**Published:** 2016-05-05

**Authors:** Regina WS Sit, Vincent CH Chung, Kenneth D. Reeves, David Rabago, Keith KW Chan, Dicken CC Chan, Xinyin Wu, Robin ST Ho, Samuel YS Wong

**Affiliations:** 1 The Chinese University of Hong Kong, The Jockey Club School of Public Health and Primary Care, Hong Kong; 2 The University of Kansas, Department of Physical Medicine and Rehabilitation (1986-2015), Kansas City, USA; 3 University of Wisconsin School of Medicine and Public Health, Department of Family Medicine and Community Medicine, Madison, Wisconsin, USA.

## Abstract

Hypertonic dextrose injections (prolotherapy) is an emerging treatment for symptomatic knee osteoarthritis (OA) but its efficacy is uncertain. We conducted a systematic review with meta-analysis to synthesize clinical evidence on the effect of prolotherapy for knee OA. Fifteen electronic databases were searched from their inception to September 2015. The primary outcome of interest was score change on the Western Ontario and McMaster Universities Arthritis Index (WOMAC). Three randomized controlled trials (RCTs) of moderate risk of bias and one quasi–randomized trial were included, with data from a total of 258 patients. In the meta-analysis of two eligible studies, prolotherapy is superior to exercise alone by a standardized mean difference (SMD) of 0.81 (95% CI: 0.18 to 1.45, p = 0.012), 0.78 (95% CI: 0.25 to 1.30, p = 0.001) and 0.62 (95% CI: 0.04 to 1.20, p = 0.035) on the WOMAC composite scale; and WOMAC function and pain subscale scores respectively. Moderate heterogeneity exists in all cases. Overall, prolotherapy conferred a positive and significant beneficial effect in the treatment of knee OA. Adequately powered, longer-term trials with uniform end points are needed to better elucidate the efficacy of prolotherapy.

Knee osteoarthritis (OA) is the most common form of chronic arthritis worldwide and a major cause of pain and disability^1^,^2^,^3^. It carries considerable economic burden with its high prevalence, loss of work time and utilization of healthcare service resources^4^,^5^. The effectiveness of current treatment for KOA is limited. Current guidelines suggest that the management of knee OA should take a multidisciplinary approach including non-pharmacological, pharmacological, surgical and complementary therapies^6^,^7^. Despite efforts by the Osteoarthritis Research Society International (OARSI) Treatment Guidelines Committee, there are limited generally accepted core sets of treatments for patients with knee OA^8^. A safe and effective treatment option that complements the current therapy remains a top priority in clinical practice and research. In the United States, assessment of knee OA treatment has been identified as a “top 100” research priority by the National Academy of Medicine^9^, and the Agency for Healthcare Research and has called for new knee OA therapy^10^.

Hypertonic dextrose injection, “prolotherapy”, is an injection-based treatment used for a variety of painful chronic musculoskeletal pain conditions, including knee OA^11^. The core practice principle of prolotherapy is injection of relatively small volumes (0.5–6 ml) of an irritant solution, usually hypertonic dextrose, at painful ligament and tendon attachments, as well as in adjacent joint spaces^11^. The basic science of the mechanism of action on connective tissue and cells of various type (e.g. fibroblast/chondrocytes etc.) is not well understood. However, animal studies have reported that peritendinous dextrose injection consistently resulted in fibroblast and vascular proliferation, dense collagen deposition and increase in ligament thickness, energy absorption and ultimate load bearing ability^12^,^13^,^14^,^15^. Animal model data has also suggested cartilage-specific anabolic growth as a result of intra-articular dextrose injection^16^. The hypothesized mechanisms for pain relief include: (i) stimulation of local healing among chronically injured extra- and intra-articular tissue; (ii) reduction of joint instability through the strengthening of stretched or torn ligaments, and (iii) stimulation of cellular proliferation^17^.

Prolotherapy is practiced throughout the world; the strongest interest appears to be among physicians and patients in primary care^18^,^19^. Human studies have assessed the role of prolotherapy for various musculoskeletal conditions^20^,^21^,^22^,^23^,^24^. Systematic reviews of the clinical effectiveness of prolotherapy in chronic low back pain and lateral epicondylitis had been conducted in the past which yielded mixed and positive results respectively, though the strength of evidence was limited by clinical heterogeneity amongst studies and the presence of co-interventions^25^,^26^. In recent years, prolotherapy has been used to treat patients with knee OA refractory to other conservative care^27^. Results from several published clinical trials have shown positive effects of prolotherapy in knee OA but the findings have not been synthesized^28^,^29^,^30^,^31^. Therefore, we conducted a systematic review with the aim of more comprehensively assessing the efficacy of prolotherapy for knee OA in order to clarify its potential role as a non-surgical treatment modality.

## Methods

We follow the PRISMA Reporting Guidelines for Systematic Review and Meta-analysis^32^:

### Search methods for identification of studies

Potential studies were identified by searching the electronic databases listed below, with search period starting from their inception till September 2015 (Appendix 1).

Cochrane Central Register of Controlled Trials (CENTRAL)

OVID MEDLINE

EMBASE

Global Health,

NHS Health Technology Assessment Database,

Digital Dissertation Consortium,

International Pharmaceutical Abstract

BIOSIS Preview.

AMED

Inspec

Ovid Nursing Database

CinicalTrial.gov (http://clinicaltrials.gov/),

Drugs@FDA (http://www.accessdata.fda.gov/scripts/cder/drugsatfda/index.cfm),

European Medicines Agency public assessment reports (EPAR, http://www.ema.europa.eu/ema),

Pharmaceuticals and medical devices agency of Japan (http://www.pmda.go.jp/english/service/approved.html).

### Types of studies

This systematic review included randomized controlled trials (RCTs) and quasi-randomized controlled trials that compared prolotherapy injections to saline injections, water injections or exercise therapy. Co-interventions were allowed, as long as they were uniform across all groups. There were no limitations on publication dates. The protocol was registered at PROSPERO (CRD42015015901).

### Types of participants

We selected studies that included participants aged 18 years or over with (i) a diagnosis of knee OA according to the criteria from the American College of Rheumatology^33^, (ii) knee pain for at least 3 months and (iii) had reported the Western Ontario and McMaster Universities Arthritis Index (WOMAC)^34^ or the Visual Analogue Scales (VAS) for pain as one of the outcomes^35^. We excluded studies that included participants who had undergone total knee replacement.

### Types of intervention

For inclusion, dextrose prolotherapy injections had to be administered to at least one group within the trial. For comparison groups we included studies that provided injections with 0.9% normal saline or water; or exercise therapy. Consistent with the clinical practice of prolotherapy, at least part of the injection protocol had to include an intra-articular injection, with or without additional injections to the peri-articular ligament or tendon attachments.

### Outcome measures

In accordance with international consensus on the core set of outcome measures for phase III clinical trials in OA, eligible trials needed to include assessment of either self- reported pain or self-reported physical function^36^. Following this recommendation, the primary outcome of interest is the Western Ontario and McMaster Universities Arthritis Index (WOMAC)^37^. Secondary outcomes of pain include the Visual Analogue Scales (VAS) for pain^35^, Knee Pain Scale (KPS)^38^ and Wong-Baker Scale^39^.

### Eligibility Assessment and Data Extraction

Two reviewers (RS and VC) independently screened electronically retrieved titles and abstracts, evaluated potentially relevant full texts, and determined study eligibility. For eligible studies, data were extracted independently by the two authors (RS and DC) using a piloted data extraction form. For each eligible study, the following data were extracted: study design, clinical settings, participant characteristics, features of interventions, outcomes, duration of follow up and adverse events. An attempt was made to contact study authors regarding these methodological elements if not reported.

### Risk of bias assessment

The risk of bias among included studies was assessed by the Cochrane’s risk of bias tool^40^ by two reviewers independently (XYW and RH). The following risk of bias domains were evaluated: sequence generation, allocation concealment, blinding of participants and research personnel, blinding of outcome assessors, incomplete outcome data, and selective outcome reporting. Discrepancies in study selection, data extraction and risk of bias assessment results were resolved by group consensus. In the group consensus process, the two reviewers discussed reasons for discrepancy with a goal to achieve consensus after clarification. If a consensus could not be reached, a third reviewer Vincent Chung (VC) was included as an arbitrator. A decision was made after reevaluation of the included studies and further discussion between the three reviewers. This has been clarified in the revised manuscript.

### Statistical Analysis

All meta-analyses were conducted using the STATA software^41^. A random effect model was used to pool study results, taking into account possible variations in effect sizes across trials^42^. Changes in continuous outcomes were pooled as standardized mean differences (SMD), as different scaling of outcome measurements across trials were expected. 95% confidence intervals (CI) were calculated for all estimates. I square (I^2^) statistic was calculated to estimate heterogeneity across studies. An I^2^ level of less than <25%, 25–50% and greater than 50% were regarded as indicators of low, moderate and high levels of heterogeneity respectively^43^.

### Minimal Clinically Important Difference (MCID) for WOMAC and VAS

The effect estimates were interpreted according to the values of the established Minimal Clinically Important Difference (MCID). It has been reported that a 12-point increment on WOMAC composite scale indicates ‘good to very good’ improvement when the scale is transformed onto a 0–100 point scale^44^,^45^.

The MCID for VAS global pain assessment score and VAS pain on motion were based on a study in 2005; of which the MCID for improvement in the global VAS is 18.3 mm whereas that for VAS in motion is 19.9 mm^44^.

## Results

**Figure 1:**
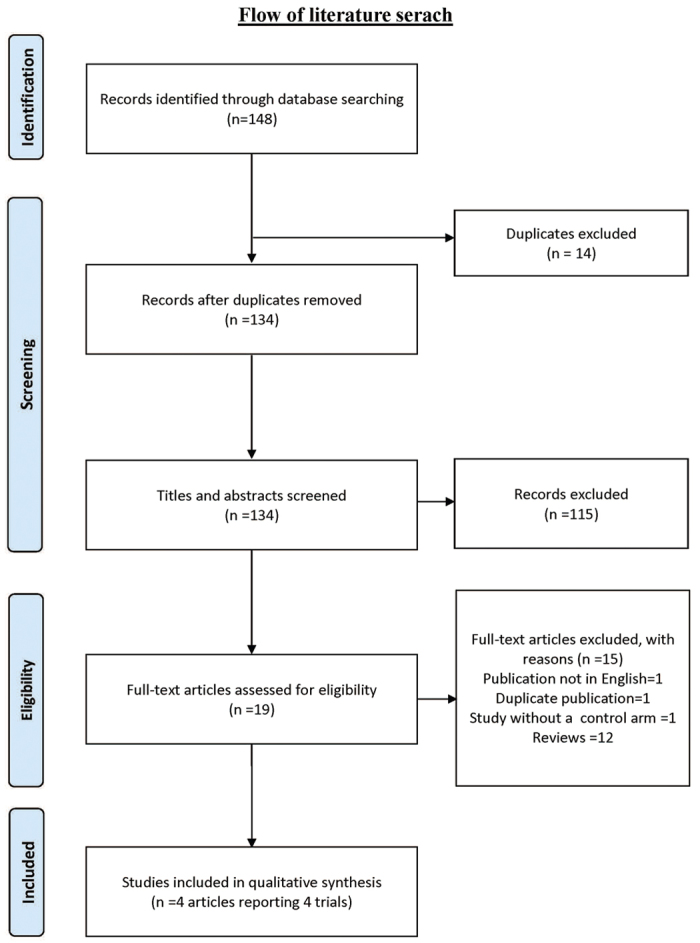
Flow of literature search.

We identified 134 citations from all searches and excluded 14 duplicates. After screening the titles and abstracts, we retrieved 19 full texts for further assessment. Of these, 15 were excluded for the following reasons: duplicate publication as conference abstract (n = 1), publication not in English (n = 1), trial without a control arm (n = 1) and narrative reviews (n = 12). Four full texts which reported results from four clinical trials were eligible for inclusion^28^,^29^,^30^,^31^.(Fig. 1)

### Characteristics of included trials

Characteristics of included trials are summarized (Table 1). The four included clinical trials^28^,^29^,^30^,^31^ recruited a total of 258 patients with a diagnosis of knee OA based on American College of Rheumatology (ACR) guidelines including baseline radiological severity as graded by a Kellgren-Lawrence (K-L) score (Table 2)^46^. The follow up period ranged from five weeks to one year post-enrollment. The injection protocols varied; two studies referred to an existing published injection protocol^47^.

### Risk of bias

Risk of bias amongst included studies was moderate overall (Table 3). None provided a detailed publicly accessible protocol of study methods including planned a *priori* statistical analysis methods (e.g. study protocols on trial registration websites such as ClinicalTrials.gov) that allowed assessment of selective outcome reporting, but three of them have provided a detailed analysis plan via personal communication^29^,^30^,^31^. Sequence generation was considered to have a low risk of bias in only two studies, and two had a low risk of bias in allocation concealment. Two studies blinded both patients and investigators, but two did not. Two studies reported blinding of assessors but not the remaining two. Risk of bias incurred from incomplete outcome data varied, with drop-out rates ranged from 0% to 27.8%. Since three out of four trials did not provide trial protocol, this can be regarded as a major source of bias.

### Effect of interventions

The effect of interventions with different comparison groups and follow up duration is summarized in Table 4. Dumais *et al.*^28^ used WOMAC version constructed on a five point (0–4) Likert scale: composite (0–96), pain (0–20), stiffness (0–8) and function (0–68). Lower score indicated better knee related outcomes. Rabago *et al.*^30^,^31^ used a WOMAC version constructed on the same five point (0–4 point scale) Likert scale. Each of the individual subscales was then converted to a 0–100 score scale, with higher score reflecting improvement.

### Dextrose Injection versus Exercise on WOMAC index

**Figure 2:**
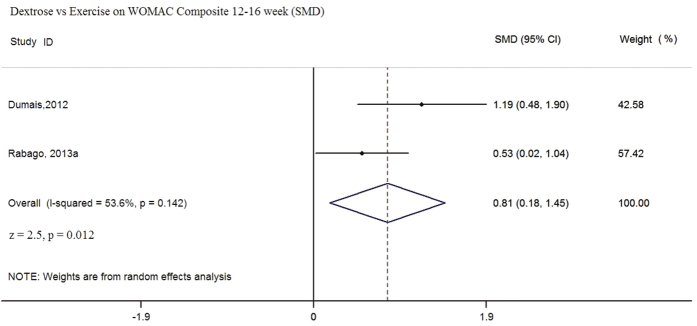
Dextose vs Exercise on WOMAC Composite 12–16 week (SMD).

**Figure 3:**
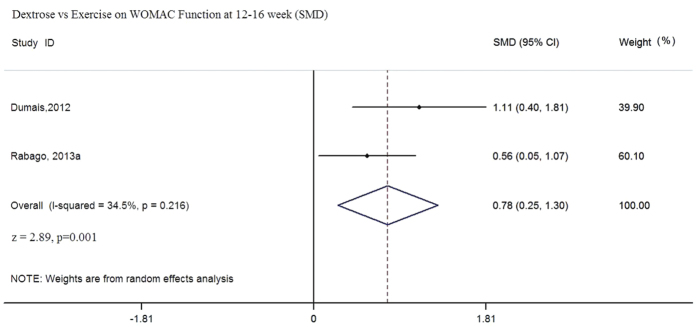
Dextrose vs Exercise on WOMAC Function at 12–16 week (SMD).

**Figure 4:**
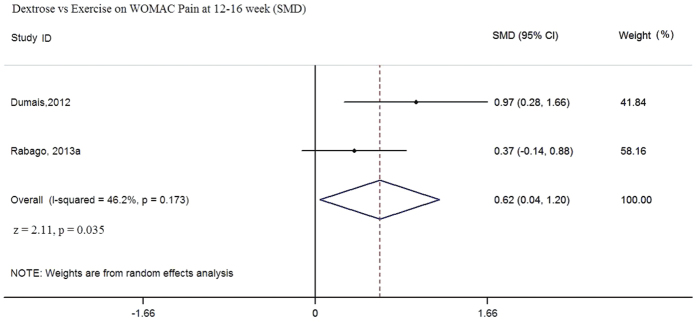
Dextrose vs Exercise on WOMAC Pain at 12–16 week (SMD).

In this comparison, two RCTs (n = 97) were eligible for pooling using follow-up data from 12 to 16 weeks^28^,^30^. Although the same WOMAC tool was used, different measurement scales were adopted in these two trials; therefore, SMDs were calculated in the random effect meta-analyses. Pooled results favored prolotherapy in terms of WOMAC composite and WOMAC function scores (Figs 2 and 3). The pooled SMD of WOMAC composite score is 0.81 (95% CI: 0.18 to 1.45, p = 0.012, I^2^ = 53.6%); whereas the SMD of WOMAC-function score is 0.78 (95% CI: 0.25 to 1.30, p = 0.001, I^2^ = 34.5%). For WOMAC pain (Fig. 4), pooled results indicated that the SMD is 0.62 (95% CI: 0.04 to 1.20, p = 0.035, I^2^ = 46.2%). While the p value indicates statistically significant result as defined by p < 0.05, the lower end of the 95% CI is very close to zero, suggesting the imprecision of possible treatment effect. Overall, sizes of the SMD indicated that dextrose injection provides substantially stronger improvement when compared to exercise, but the lower end of 95% CI has covered SMD values of small effect sizes (i.e. SMD ≤ 0.2)^48^. Such uncertainty is also reflected in the low to moderate degree of heterogeneity among all three meta-analyses, given all with I^2^ values ranging from 34.5% to 53.6%^49^. Although it is not included in meta-analysis due to differences in data collection time, Rabago *et al.*^22^ also reported WOMAC outcomes at 26 and 52 week follow-up. Dextrose injections outperformed exercise on the composite and function-subscale score (26 and 52 weeks) and the pain-subscale score (26 weeks) respectively^30^.

### Dextrose versus Saline Injection on WOMAC index at 12 weeks and 52 weeks

In two trials, dextrose injections, either on their own^30^ or mixed with sodium morrhuate^31^, were found to be superior to normal saline in improving WOMAC composite and subscale scores to levels above the minimal clinical important difference (MCID) at 12 and 52 weeks^35^. (Table 4)

### Pain intensity on VAS at 16 and 24 weeks after prolotherapy

At 16 weeks, Dumais *et al.* reported an improvement in the VAS global pain score of 29.7 points (SE: 4.57), above the MCID at 24 weeks, Reeves *et al.* also reported improvement in VAS pain during walking of 13.9 (SE: 3.10) and during stair climb of 13.7 (SE 3.2)^29^, which were slightly lower than the relevant MCID^44^. Pooling of the two trials was not possible as the VAS measured slightly different outcomes; the former represented a global assessment of pain whereas the latter measured pain level during movement.

### Change in pain severity at 16 weeks and 24 weeks after prolotherapy

At 16 week, Dumais *et al.* reported statistically significant improvement in Wong Baker Scale scores after prolotherapy (adjusted mean change: 1.27, SE: 0.33, p = 0.003)^28^. At 24 weeks, Rabago *et al.*^22^ also reported a statistically significant improvement in WOMAC pain scores (adjusted mean change 15.50 ± SE 3.56, p < 0.05) and on the KPS (adjusted mean change: 0.92, SE: 0, 25, p < 0.05). The pain reduction magnitude of active participants was significantly greater when compared with their control groups (p < 0.05)^30^.

### Side effects

All four trials monitored side effects and adverse effects. Only one trial reported self-limited bruises after both dextrose (n = 3) and saline injections (n = 5)^30^. This was an expected side effect and deemed to be of minimal clinical relevance due to its transient nature

## Discussion

Pooling data from two RCTs, we report that peri- and intra-articular hypertonic dextrose knee injections in three to five sessions have a statistically significant and clinically relevant effect in the improvement of WOMAC composite score, functional and pain subscale at 12 to 16 weeks compared to formal at-home exercise. Self-reported outcomes favoring prolotherapy are also observed in unpooled data when dextrose prolotherapy groups are compared with other control groups. The majority of the effect sizes are higher than the MCID, and the benefits were sustained up to 1 year. The risk of bias level in the included studies was moderate. Overall, prolotherapy injections appear to be safe, but no study was powered to detect rare adverse events.

While the overall direction of the effect is positive, some uncertainty of the effect size still exists due to the low to moderate heterogeneity and wide confidence intervals. In addition, this systematic review identified several limitations of existing trials. None of the included studies provided a publicly accessible protocol, therefore the potential for selective outcome reporting exists; however, the analyses are generally straightforward and well-reported. The sample size was small with only four trials eligible for review and only portions of two trials could be pooled in meta-analyses. Small sample size led to a wide confidence interval; thus the effect sizes reported may be imprecise. However, pooled results provide guidance for the sample size needed in future clinical studies^50^. Due to a lack of uniform longer-term follow up data across both studies, pooling of results could only be done with data collected between 12 to 16 week follow-up. Given that prolotherapy is hypothesized to work by healing and regeneration over several months^51^,^52^, the effects reported here may underestimate longer term benefits. A recent open label study reported progressive improvement in WOMAC scores for most study participants through 2.5 ± 0.6 years (range 1.6–3.5 years) of follow up after prolotherapy^27^. Finally, low to moderate quantified heterogeneity was reported in pooled analyses; this is likely attributable to multiple factors, including differences in patient characteristics, control treatment, study design, injection protocol methods, dextrose concentration, follow-up duration and outcomes assessment methods.

While the positive findings in the evaluated studies suggest efficacy, their limitations restrict a rigorous determination of efficacy and clinical utility. The current analysis suggests studies with the following characteristics can be considered in the future: 1) study duration of at least one year. Prolotherapy is hypothesized to be a regenerative injection therapy; serial monthly injections are typically performed to stimulate hypothesized tissue effects; 2) uniform self-reported outcomes and time points using the WOMAC outcome measures^37^; 3) objectively determined physical function and health status outcomes such as hospitalization, nursing home placement and decline in health^53^,^54^. Inclusion of objective assessment including magnetic resonance imaging^31^ and intra-articular and serum biomarkers^55^. 4) a *priori* subgroup analysis to identify the phenotype of patients who respond most favorably to prolotherapy;5) because the performance of prolotherapy appears to be superior to saline and exercise, future studies may consider formal effectiveness designs using exercise or other non-injection matched control comparators. This avoids injection-related discomfort and risk among controls, saves study conduct time and cost, and offers a comparison relevant to clinical practice; 6) overall quality of life and cost effectiveness evaluation using the EuroQol-5D, a commonly used measure that allows comparison to other interventions^56^; 7) determination of the MCID of WOMAC score specific to prolotherapy.

Investigation of prolotherapy is in an early stage and several treatment protocol issues warrant further study to determine optimal strategy, including dextrose concentration and volume, treatment frequency and duration, and specific utility of intra compared with extra-articular injections. Studies in this review used the approach of dextrose injections into the intra-articular joint space and the extra-articular soft tissue attachment. Recently, more superficial application of hypertonic dextrose has been reported to provide an analgesic effect in the treatment of a variety of peripheral neuropathic pain conditions^57^,^58^. Sensorineural effects of dextrose have been hypothesized^31^. Given that the etiology of knee OA pain is multifactorial, future trials may consider adding superficial dextrose injection as part of the protocol. Concomitant basic science studies using *in vitro* and animal model methodologies are warranted to better elucidate the mechanism of action of dextrose in the context of musculoskeletal pathology. Finally, studies in this review used palpation guidance for injections; future investigators should also consider the use of ultrasound guidance to improve injection accuracy and precision, limiting procedural variability^59^.

### Strengths and weaknesses of this systematic review

The main limitation of this systematic review is the limited number of studies and their relatively small sample size. We considered whether or not to perform a meta-analysis in addition to systematic narrative review, given that the small sample sizes of included trials might generate an unstable pooled effect size^60^. A meta-analyses is included and resulted in increased statistical rigor by increasing the overall sample size, providing a more precise estimate^61^. In a narrative review, evaluation of efficacy in RCTs is based primarily on the p-values of individual studies, a so-called ‘vote counting’ approach, and the risk of arriving at a biased conclusion may even be higher^62^. Because most of the results in the included trials were positive, readers may in the case of narrative review be tempted to draw an over-confident conclusion^63^. A meta-analysis, with calculation of I^2^ value, is able to express current best evidence and heterogeneity among studies quantitatively^64^. Specifically, we determined that the I^2^ value of WOMAC composite score is 53.6%, indicating considerable heterogeneity on the effect estimate, a finding that can be communicated only through meta-analysis^65^.

Strengths include the conduct of a comprehensive literature search, duplicate study selection and data extraction by two independent reviewers, and appraising risk of bias of all included studies using a validated tool independently by two reviewers.

## Conclusion

The results of this systematic review indicate that hypertonic dextrose prolotherapy conferred a positive, significant beneficial effect meeting criteria for clinical relevance in the treatment of knee OA, compared with saline injection and exercise. However, moderate heterogeneity existed among these trial results. Larger, long-term trials with uniform outcomes and high methodological standards are needed for more a more comprehensive assessment of the overall treatment effect of prolotherapy.

## Additional Information

**How to cite this article**: Sit, R. W. S. *et al.* Hypertonic dextrose injections (prolotherapy) in the treatment of symptomatic knee osteoarthritis: A systematic review and meta-analysis. *Sci. Rep.* **6**, 25247; doi: 10.1038/srep25247 (2016).

## Supplementary Material

Supplementary Information

## Figures and Tables

**Table 1:** Characteristics of included studies.

Source & no. of arms	Intervention	Injection route and solution concentration (%)	Total no of participants	Number of case analyzed	Age +/− standard deviation	Sex Female (%)	Weight +/− standard deviation	Body mass index (BMI)	No of injections received +/−standard deviation	Outcomes assessed	Trial duration (Time for end points assessment)
Rabago^30^ (2 arms)	Dextrose OR Dextrose plus Morrhuate	Intra-articular dextrose 25%, Extra-articular dextrose 15%, Sodium Morrhuate 5%	27	24	54.5+/−6.8	54.2	Not reported	25% of the subjects with BMI ≦25 37.5% of the subjects with BMI 26–30 37.5% of the subjects with BMI ≧31	4.5+/−0.9		52 weeks
										WOMAC composite -WOMAC-pain -WOMAC-stiff -WOMAC-function	(week 5,9,12,24,52)
	Saline OR Exercise	Intra-articular and extra-articular saline 0.9%	18	13	57.4+/−7.6	61.5	Not reported	30.8% of the subjects with BMI ≦25, 30.8% of the subjects with BMI 26–30, 38.5% of the subjects with BMI ≧31	4.3+/−0.8	Cartilage volume	(at week 52 only)
Rabago^22^ (3 arms)	Dextrose	Intra-articular dextrose 25%, Extra-articular dextrose 15%,	33	30	56.8+/−7.9	63	Not reported	33% of the subjects with BMI ≦25, 20% of the subjects with BMI 25–30, 47% of the subjects with BMI, ≧30	3.95+/−1	WOMAC composite WOMAC-pain WOMAC-stiff WOMAC-function KPS (each knee)	52 weeks (week 5,9,12,24,52)
	Saline	Intra-articular and extra-articular saline 0.9%	31	29	56.8+/−6.7	69	Not reported	28% of the subjects with BMI ≦25, 38% of the subjects with BMI 25–30, 34% with BMI ≧30	3.71+/−1.1		
	Exercise	N/A	33	31	56.4+/−7.0	68	Not reported	19% of the subjects with BMI ≦25, 39% of the subjects with BMI 25–30, 42% of the subjects with BMI ≧30	N/A		
Dumais *et al.*^28^ (2 arms with cross over)	Dextrose	Intra-articular dextrose 20%, Extra-articular dextrose 15%	21	18	57.3+/−12.6	38.9	90.1 (22)	32.2+/−7.2	3.7+/−0.7	WOMAC composite -WOMAC-pain -WOMAC-stiff -WOMAC-function Pain intensity	32 weeks (data collected at week 16 before cross- over; then reassessed at week 32)
	Exercise	N/A	24	18	56.2+/−10.9	55.6	92.4 (17.2)	34.3+/−5.7	N/A	Functional impairment Wong-Baker Descriptive Numerical VAS Combined pain score Timed UGT	
Reeves *et al.*^21^ (2 arms)	Dextrose	Intra-articular dextrose10%	36	35	60.0+/−12.5	51.4	86.6 (19.2)	Not reported	3	VAS- rest VAS- walking VAS-stair use Swelling Buckling/2 mon Flexion range	24 weeks (assessed at week 24 only)
	Water	Intra-articular bacteriostatic water	35	32	65.3+/−11.0	34.3	90.5(19.1)	Not reported	3		

KEY:

BMI = Body Mass Index.

VAS = Visual Analogue Scale.

UGT = Up and Go Test.

WOMAC = Western Ontario and McMaster Universities Arthritis Index.

KPS = Knee Pain Scale.

**Table 2:** Distribution of OA knee severity grading among participants of included trials.

Study	Interventions and controls	Kellgren Lawerence Grade 2 or above	
Reeves 2010	Dextrose	36	
	Normal saline	25	
		Kellgren-Lawerenc Grade 1 or 2(mild)	Kellgren-Lawerence grade 3 or 4 (moderate to severe)
Dumais^28^	Dextrose	3	15
	Exercise	2	16
Rabago^22^	Dextrose	11	14
	Normal Saline	12	9
	Exercise	9	14
Rabago^30^	Dextrose (Dextrose alone or dextrose + morrhuate) & Control (normal saline or exercise)	157	96

**Table 3:** Risk of bias assessment.

Source	Sequence generation	Allocation concealment	Blinding of participants and researchers	Blinding of outcome assessment	Incomplete outcome data addressed	Selective outcome reporting
Reeves^21^	Low. Random sequence was generated by random number table.	Unclear. Relevant information was not reported.	Low. Identical control solution was used	Low. Films were separated in different packets so reading 1 film would not influence reading of the next. X ray films were read by the chief investigator. A database coordinator loaded results onto the database.	High. Lost to follow up was 9/77 = 11.7% > 10%	Unclear. No protocol was provided.
Dumais^28^	Unclear. Authors did not provide information on how random sequence was generated.	Low. Opaque sealed envelopes were used.	High. It was an open-labeled trial.	High. It was an open-labeled trial.	High. Lost to follow up was 3/21 = 14.3% in group A. 6/24 = 25% in group B. Both groups >10%	Unclear. No protocol was provided.
Rabago^22^	Low. Random sequence was generated by computer.	Low. Allocation concealment of injectant was achieved through the use of sealed opaque envelopes (personal communication, Rabago 2015)	Low. Both active and control solutions looked similar.	Low. The injector, outcome assessor, principal investigator, and participants were blinded to injection group by preparation of syringes off site; blinding was formally assessed among injection participants and injector using questionnaire.	Low. No lost to follow-up cases.	Low. No protocol was provided,but analysis plan was clearly described.
Rabago^30^	High. Non-randomized study design.	High. Non-randomized study design.	High. Single-arm uncontrolled study/at-home exercise “control” was used.	High. Primary outcome was self-reported QOL.	High. Lost to follow up was 3/27 = 11.1% in intervention group. 5/18 = 27.8% in control group. Both >10%	Unclear. No protocol was provided.

**Table 4:** Effectiveness of prolotherapy injection by comparison type for WOMAC composite, WOMAC pain, WOMAC Function scores and pain intensity.

	**Outcome: WOMAC Composite at 12–16 week**							
	Comparison	n	Scale Range ^	Pre-treatment mean score	Standard deviation	Post-treatment adjusted mean change	Standard error	Duration of follow up
Dumais^28^	Dextrose	18	0–96	44.4	13.7	−21.8 *€*	2.95	16 week
	Exercise	18	0–96	36.2	16.8	−6.1 *€*	3.28	
Rabago^22^	Dextrose	30	0–100	63.1	15.0	13.31	3.32	12 week
	Normal saline	29	0–100	62.7	14.3	8.19	3.37	
	Exercise	31	0–100	60.5	11.3	4.26	3.36	
Rabago^30^	Dextrose (dextrose alone or dextrose + morrhuate)	24	0–100	59.9	12.2	14.2	2.7	12 week
	Control (normal saline or exercise)	13	0–100	67.0	10.8	7.0	3.4	
	**Outcome: WOMAC Pain at 12–16 week**							
Dumais^28^	Dextrose	18	0–20	9.5	2.9	−5.0 *€*	0.78	16 week
	Exercise	18	0–20	8.7	4.0	−1.9 *€*	0.73	
Rabago^22^	Dextrose	30	0–100	66.8	14.9	11.78	3.62	12 week
	Normal saline	29	0–100	66.7	16.1	5.79	3.67	
	Exercise	31	0–100	63.2	13.1	4.89	3.66	
Rabago^30^	Dextrose (dextrose alone or dextrose + morrhuate)	24	0–100	62.3	12.9	15.4	3.0	12 week
	Control (normal saline or exercise)	13	0–100	71.9	13.5	3.5	3.9	
	**Outcome: WOMAC Function at 12–16 week**							
Dumais^28^	Dextrose	18	0–68	33.6	10.7	−14.6 *€*	2.14	16 week
	Exercise	18	0–68	26.8	12.8	−3.6 *€*	2.52	
Rabago^22^	Dextrose	30	0–100	65.2	15.8	14.61	3.40	12 week
	Normal saline	29	0–100	67.6	17.5	6.63	3.44	
	Exercise	31	0–100	61.9	12.7	4.89	3.43	
Rabago^30^	Dextrose (dextrose alone or dextrose + morrhuate)	24	0–100	62.6	12.9	14.3	2.8	12 week
	Control (normal saline or exercise)	13	0–100	68.5	11.3	7.8	2.8	
	**Outcome: pain intensity at 16–24 week (measured on a Visual Analogue scale VAS of 0–100 mm)**							
Dumais^28^ #	Dextrose	18	0–100	48.6	21.8	−29.70 *€*	4.57	16 week
	Exercise	18	0–100	38.3	24.8	−9.92 *€*	4.58	
Reeves 2002$	Dextrose	36	0–100	21.5	22.4	−5.4	2.4	24 week
	Water	35	0–100	27.3	20.2	−10.4	2.5	
Reeves 2002^£^	Dextrose	36	0–100	39.4	28.2	−13.9	3.1	
	Water	35	0–100	38.3	22.0	−9.80	3.2	
Reeve 2002^&^	Dextrose	36	0–100	53.3	28.0	−13.7	3.2	
	Water	35	0–100	58.3	26.0	−12.3	3.2	
	**Outcome: pain intensity at 16–24 week (Wong Baker/Knee pain scale)**							
Dumais^28^ *	Dextrose	18	WBS 0–5	2.7	1.2	−1.27 *€*	0.33	16 week
	Exercise	18	WBS 0–5	2.3	1.1	−0.19 *€*	0.26	
Rabago^22^ ¥	Dextrose	43	KPS 0–5	1.8	0.8	−0.92	0.25	24 week
	Normal saline	41	KPS 0–5	1.7	0.7	−0.26	0.25	
	Exercise	47	KPS 0–5	1.7	0.8	−0.33	0.24	
	**Outcome: WOMAC Composite at 52 week**							
Rabago^22^	Dextrose	30	0–100	63.1	15.0	15.32	3.32	
	Normal saline	29	0–100	62.7	14.3	7.59	3.36	
	Exercise	31	0–100	60.5	11.3	8.24	3.33	
Rabago^30^	Dextrose (dextrose alone or dextrose + morrhuate)	24	0–100	59.9	12.2	17.6	3.2	
	Control (normal saline or exercise)	13	0–100	67.0	10.8	8.6	5.0	
	**Outcome WOMAC Pain at 52 week**							
Rabago^22^	Dextrose	30	0–100	66.8	14.9	14.18	3.62	
	Normal saline	29	0–100	66.7	16.1	7.38	3.67	
	Exercise	31	0–100	63.2	13.1	9.24	3.63	
Rabago^30^	Dextrose (dextrose alone or dextrose + morrhuate)	24	0–100	62.3	12.9	18.1	3.8	
	Control (normal saline or exercise)	13	0–100	71.9	13.5	4.6	5.0	
	**Outcome WOMAC function at 52 week**							
Rabago^22^	Dextrose	30	0–100	65.2	15.8	16.25	3.39	
	Normal saline	29	0–100	67.6	17.5	5.46	3.44	
	Exercise	31	0–100	61.9	12.7	7.31	3.40	
Rabago^30^	Dextrose (dextrose alone or dextrose + morrhuate)	24	0–100	62.6	12.9	18.6	2.9	
	Control (normal saline or exercise)	13	0–100	68.5	11.3	9.8	4.8	

Keys:

^#^The VAS score in Dumais 2012 represents a global assessment of disease activity, with minus sign (−) indicating improvement.

^$^The VAS score in Reeves 2002 represents measurement of pain intensity at rest, with minus sign (−) indicating improvement.

^£^The VAS score in Reeves 2002 represents measurement of pain intensity with walking, with minus sign (−) indicating improvement.

^&^The VAS score in Reeves 2002 represents measurement of pain intensity with stair use, with minus sign (−) indicating improvement.

^*^Wong Baker = Wong Baker Faces Pain Rating scale from 0–5, with minus sign (−) indicating improvement.

^¥^The Knee pain scale assessed severity on a 0–5 ordinal scale on each individual knee, with minus sign (−) indicating improvement.

^^^Dumais adopted WOMAC version constructed on a five point Likert scale: composite (0–96), pain (0–20), stiffness (0–8) and function (0–68). Lower score indicates better knee related metrics, minus sign (−) indicates pain reduction.

^^^Rabago adopted WOMAC version constructed on a five point Likert scale: composite (0–96), pain (0–20), stiffness (0–8) and function (0–68); values were then converted to a 0–100 scale for each of the four domains. Composite WOMAC score reflect a weighted average. Higher score indicates better knee related metrics, of which a positive sign (+) indicates improvement.

^€^The values in standard deviation were converted to standard error.
