# Erratum: Hypertonic dextrose injections (prolotherapy) in the treatment of symptomatic knee osteoarthritis: A systematic review and meta-analysis

**DOI:** 10.1038/srep45879

**Published:** 2017-04-07

**Authors:** Regina WS Sit, Vincent CH Chung, Kenneth D. Reeves, David Rabago, Keith KW Chan, Dicken CC Chan, Xinyin Wu, Robin ST Ho, Samuel YS Wong

Scientific Reports 6: Article number: 2524710.1038/srep25247; published online: 05 05 2016; updated: 04 07 2017

This Article contains errors in Tables 1, 2, 3 and 4 where references 29, 30 and 31 are incorrectly given as references 21, 22 and 30 respectively.

