# Supplementary material for: Hypertonic dextrose injections (prolotherapy) in the treatment of symptomatic knee osteoarthritis: A systematic review and meta-analysis
Source: Sci Rep. 2016 May 5;6:25247. doi: 10.1038/srep25247 (PMC4857084; doi:10.1038/srep25247)
Supplement: Supplementary Information [file srep25247-s1.pdf]

Hypertonic dextrose injections (prolotherapy) in the treatment of symptomatic knee osteoarthritis: A systematic review and meta-analysis

Regina WS Sit[1], Vincent CH Chung[1,*], Kenneth D. Reeves[2], David Rabago[3], Keith KW Chan[1], Dicken CC Chan[1], Xinyin Wu[1], Robin ST Ho[1], Samuel YS Wong[1]

[1] The Chinese University of Hong Kong, The Jockey Club School of Public Health and Primary Care, Hong Kong
[2] Dean K. Reeves, MD, 4740 El Monte St, Roeland Park, KS 66205
[3] University of Wisconsin School of Medicine and Public Health, Department of Family Medicine, Madison, Wisconsin
[*]vchung@cuhk.edu.hk

Appendix I : Search strategies for each databases

MEDLINE
1. exp osteoarthritis/
2. osteoarthr$.tw.
3. (degenerative adj2 arthritis).tw.
4. arthrosis.tw.
5. Prolotherapy.tw.
6. (Regenerative inject$).tw.
7. (Hypertonic dextrose inject$).tw.

clinical trial.mp.
clinical trial.pt.
random:.mp. OR tu.xs.

EMBASE
1. exp osteoarthritis/
2. osteoarthr$.tw.
3. (degenerative adj2 arthritis).tw.
4. arthrosis.tw.
5. Prolotherapy.tw.
6. (Regenerative inject$).tw.
7. (Hypertonic dextrose inject$).tw.

random:.tw.
OR clinical trial:.mp.
OR exp health care quality

CENTRAL

1. exp osteoarthritis/
2. osteoarthr$.tw.
3. (degenerative adj2 arthritis).tw.
4. arthrosis.tw.
5. Prolotherapy.tw.
6. (Regenerative inject$).tw.
7. (Hypertonic dextrose inject$).tw.

Global Health, NHS Health Technology Assessment Database, Digital Dissertation Consortium, International Pharmaceutical Abstract, BIOSIS Preview. AMED, Inspec, Ovid Nursing Database

1. Prolotherapy.tw.
2. (Regenerative inject$).tw.
3. (Hypertonic dextrose inject$).tw.
